# High amplitude propagated contractions with Glycerin versus Bisacodyl: A within-subject comparison in children undergoing colonic manometry

**DOI:** 10.1111/nmo.14544

**Published:** 2023-02-27

**Authors:** Sherief Mansi, Gracielle Bahia, Dhiren Patel, Lev Dorfman, Khalil El-Chammas, Lin Fei, Chunyan Liu, Neha R. Santucci, Kahleb Graham, Ajay Kaul

**Affiliations:** 1 Division of Gastroenterology and Hepatology, Department of Pediatrics, Cincinnati Children’s Hospital Medical Center (CCHMC), Cincinnati, Ohio, USA; 2 University of Cincinnati, Cincinnati, Ohio, USA; 3 Division of Gastroenterology, Hepatology and Nutrition, Department of Pediatrics, Cardinal Glennon Children’s Medical Center, Saint Louis University School of Medicine, St Louis, Missour, USA; 4 Department of Biostatistics and Epidemiology, CCHMC, Cincinnati, Ohio, USA

**Keywords:** Bisacodyl, children, colonic manometry, constipation, fecal incontinence, Glycerin, high amplitude propagated contractions

## Abstract

**Background:**

The presence of high amplitude propagated contractions (HAPCs) measured by colonic manometry (CM) reflect an intact neuromuscular function of the colon. Bisacodyl and Glycerin are colonic stimulants that induce HAPCs and are used for the treatment of constipation. HAPCs characteristics with each drug have not been compared before. We aimed to compare the HAPC characteristics with Bisacodyl and Glycerin in children undergoing CM for constipation.

**Methods:**

This is a prospective single-center cross-over study of children aged 2–18 years undergoing CM. All patients received both Glycerin and Bisacodyl during CM. They were randomized to group A with Bisacodyl first (*n* = 22) and group B with Glycerin first (*n* = 23), with 1.5 hours in between each dose. Differences in patient and HAPC characteristics between groups were summarized using descriptive statistics and compared using Chi-square test or Wilcoxon rank sum test as appropriate.

**Key Results:**

A total of 45 patients were included. HAPCs post Bisacodyl had a longer duration of action (median of 40 vs 21.5 min, *p* <0.0001), longer propagation (median of 70 vs 60 cm, *p* = 0.02), and more HAPCs (median of 10 vs 5, *p* < 0.0001) compared Glycerin. No differences were found in the HAPC amplitude and onset of action between both medications.

## 1 | INTRODUCTION

Constipation is one of the most common conditions referred to pediatric gastroenterologists, comprising almost 25% of all visits.^1^ There is no consensus yet on the precise definition of refractory constipation but failure to respond to conventional bowel management is a generally acceptable definition.^2^ In patients with refractory constipation, colonic manometry (CM) testing is indicated to evaluate for colonic dysmotility from both functional and organic etiology.^2–4^ CM provides information on the neuromuscular integrity of the colon by evaluating colonic muscle contractions and motility by measuring intraluminal colonic pressures throughout the cleansed colon using a manometry catheter placed endoscopically or under fluoroscopy with sensors covering the entire length of the colon from cecum to sigmoid colon.^5^ One of the most easily recognized metrics on the CM is the high amplitude propagated contraction (HAPC).^6^ HAPC is defined as high amplitude contractions of 65 mmHg or more, propagating at least 30cm distally for at least 10 s, although slightly varying definitions have been described in literature.^5,7–9^ A colonic manometry study is performed using published guidelines (ANMS and NASPGHAN guidelines).^5^ Measurements during fasting, after a meal, and after colonic stimulation by stimulants to induce HAPC are done to evaluate colonic motility. The presence of HAPCs measured by CM reflect an intact neuromuscular function of the colon.^7,10^ Abnormal HAPC propagation may be indicative of segmental colonic motor disorders.^4,5^ Complete absence of HAPC indicates colonic inertia representing global colonic dysmotility.^9^

Both Bisacodyl and Glycerin have been widely used successfully in bowel regimens in patients with constipation as a method of disimpaction as well as to prevent fecal incontinence in patients with neurogenic bowel.^6,11,12^ Motility centers use Bisacodyl rather than Glycerin while performing CM as per guidelines.^5,8^ Bisacodyl stimulates sensory nerve endings to increase colonic peristalsis as well as inhibiting intestinal water absorption.^13–16^ Glycerin is a trihydroxy sugar alcohol that exerts a laxative effect when administered into the colon and has been used in antegrade enemas successfully in patients with neurogenic bowel disorders.^17^ When administered into the colon, Glycerin is thought to act as an osmotic laxative and a stimulant which produces HAPCs.^11^ A head-to-head comparison of the stimulatory effects of each drug has never been studied. In this study, we aim to compare the HAPC characteristics of Bisacodyl and Glycerin in children undergoing CM studies. In our group’s experience, patients described more cramping and abdominal pain with Bisacodyl compared to Glycerin when used in antegrade continence enemas and rectal therapy (antegrade enemas and suppositories) though both were effective. We hypothesized that Glycerin has a similar effect as Bisacodyl in inducing HAPCs during colonic manometry. Our primary outcome was to compare the effect of both medications on HAPC induction and to compare the HAPCs characteristics induced by both medications which would provide clinical guidance for the use of both medications.

## 2 | MATERIALS AND METHODS

### 2.1 | Subjects

This is a prospective single-center cross-over study which included children aged 2-18 years undergoing CM as indicated for evaluation of colonic dysmotility from January of 2020 to December of 2021 at Cincinnati Children’s Hospital Medical Center (CCHMC). The study was approved by the institutional review board (2019-1181: Glycerin HAPC). Age-appropriate consent and assent forms for use of subject data were obtained from subjects prior to data collection and analysis. We excluded patients with allergy to either drug, patients undergoing combined colonic and antroduodenal manometry as well as patients who had manometry catheter displacement compromising the quality of the study. All children with constipation, irrespective of their diagnoses, were included and served as their own controls as all the patients received both Bisacodyl and Glycerin. The cohort included children with functional constipation, Hirschsprung’s disease (syndromic and non-syndromic children), anorectal malformation, history of tethered cord repair, and joint hypermobility syndrome, although the bulk of our cohort was comprised of functional (idiopathic) constipation.

### 2.2 | Catheter placement

All colonic manometry catheters were placed endoscopically under general anesthesia by a neurogastroenterologist at CCHMC. Medical Measurement System (MMS) was used for performance and analysis of the manometry data using water perfused catheters with 8 channels (5-10 cm spaced) to 16 channels (5 cm spaced), (Laborie Medical). We used water perfused catheters for the ability to clip them with an Endo clip to the colonic mucosa to stay in place after placement. Previous multicenter studies did not show major differences between solid state vs water perfused catheters in the assessment of HAPC.^8^ None of the patients had complications during or after the study. All studies were run the day after the endoscopic placement to count for the anesthesia effect on gut motility.^18^

Anorectal manometry used was high resolution using both the Medical Measurement System as well as Manoscan^™^ Given imaging catheters. For normal anorectal manometry, the resting pressure and rectoanal inhibitory reflex had to be normal for all patients, and in the case of a cooperative patient, rectal sensation, squeeze, and defecation dynamics had to be normal as well.

### 2.3 | Sample size

We proposed that a total sample size of 32 would be justified and adequate to give the study statistical power to compare HAPCs induced by both medications due to the nature of the study as a prospective exploratory study, which is limited by the patient availability. The only study done that is comparable was a multicenter study that studied Bisacodyl and Edrophonium on HAPCs induction and characteristics, in which they used a sample size of 40.^19^ The proposed sample size of 32 is feasible to complete the study given the total number of manometry studies performed weekly. Our final sample size for the study was 45 patients.

### 2.4 | Study protocol

**FIGURE 1:**
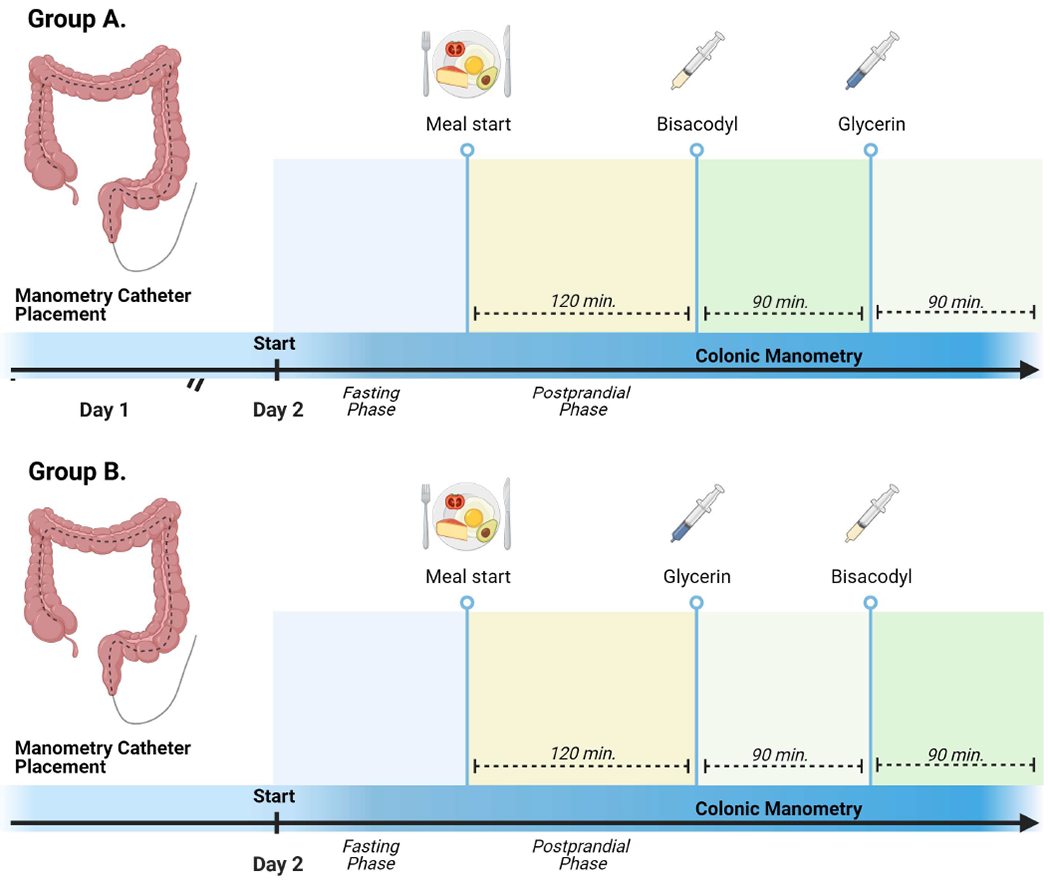
Study timeline for each patient with Group A starting Bisacodyl first and Group B starting Glycerin first.

According to the published standards for the performance of CM, all patients had recordings for at least 1 h during the fasting state and at least 2 h after a standardized meal (at least 10 kcal/kg or 400 kcal; >30% kcal from lipids).^5,20^ All patients received both Glycerin and Bisacodyl at the end of the study. They were randomized to group A with Bisacodyl first and group B with Glycerin first, with 1.5 h in between each dose. The drugs were administered intra-colonic using the central channel of the catheter (Bisacodyl dose 0.1 mg/kg with a max of 10 mg and Glycerin (concentration 1:1 with normal saline), 10 mL for patients younger than 5 years old, 20 mL for 5 years and older). See study timeline in Figure 1. The results of the colonic manometry were interpreted by 2 reviewers separately with 100% agreement.

### 2.5 | Data analysis

The data collected and analyzed after reviewing subjects’ CM tracings included presence of HAPCs during each phase (fasting, postprandial and after giving each stimulant). Furthermore, we looked at the onset of HAPC poststimulation (within 30 min of medication administration),^8,21^ number of HAPCs poststimulation, length of propagation through the colon, and duration (maximum duration allowed was 1 h postadministration, after which the effect of the stimulant typically wears off).^8^ In addition, the maximum amplitude of HAPC poststimulation was reported with each stimulant.

### 2.6 | Statistical analysis

Relevant clinical and demographic information listed, including age (at time of CM), BMI (at time of CM), gender, race, date of birth, comorbidities, anorectal manometry results, and water-soluble contrast enema (WSCE) results from electronic medical records were summarized by protocol as well as in the overall cohort. Categorical variables were described using frequency and percentages and compared using the chi-square or Fisher’s exact test (when more than 20% of the table cells have expected frequencies less than 5). The distributions of continuous variables were summarized using mean (SD), median (IQR), minimum and maximum and compared by the Wilcoxon rank sum test.

The HAPC characteristics were summarized by drugs. The distributions of the HAPC outcomes were inspected using density plots. Transformations were done if the distribution was severely skewed for the modeling. Linear mixed-effect models were used to study the drug effects on the HAPC characteristic. The independent variables included were drug, drug sequence (represent carry-over effect), and study period. Age and gender were the potential covariates. Further sequential investigations were done to evaluate whether etiology and WSCE function as independent effect modifiers through their interactions with the drug controlling for the same set of covariates as before. SAS 9.5 was used for the analysis. Statistical significance was determined if *p* <0.05.

## 3 | RESULTS

### 3.1 | Demographics and baseline characteristics

We screened 64 patients, after exclusion of 19 patients (14 denied participation, 2 had catheter displacements after placement that would interfere with data interpretation and the rest had severe pain during the study and did not strictly follow the study protocol), 45 patients were included in the study, 22 in protocol A and 23 in protocol B. The demographics are shown in Table 1. Patients in protocol A had similar characteristics as patients in protocol B in most of the variables listed in Table 1 except for age and BMI. The patients in protocol B were older (*p* = 0.02) and with higher BMI (*p* = 0.01). The mean age for the whole cohort was 10years of age (IQR 8-13) and predominantly white (91%).

### 3.2 | CM findings

**FIGURE 2:**
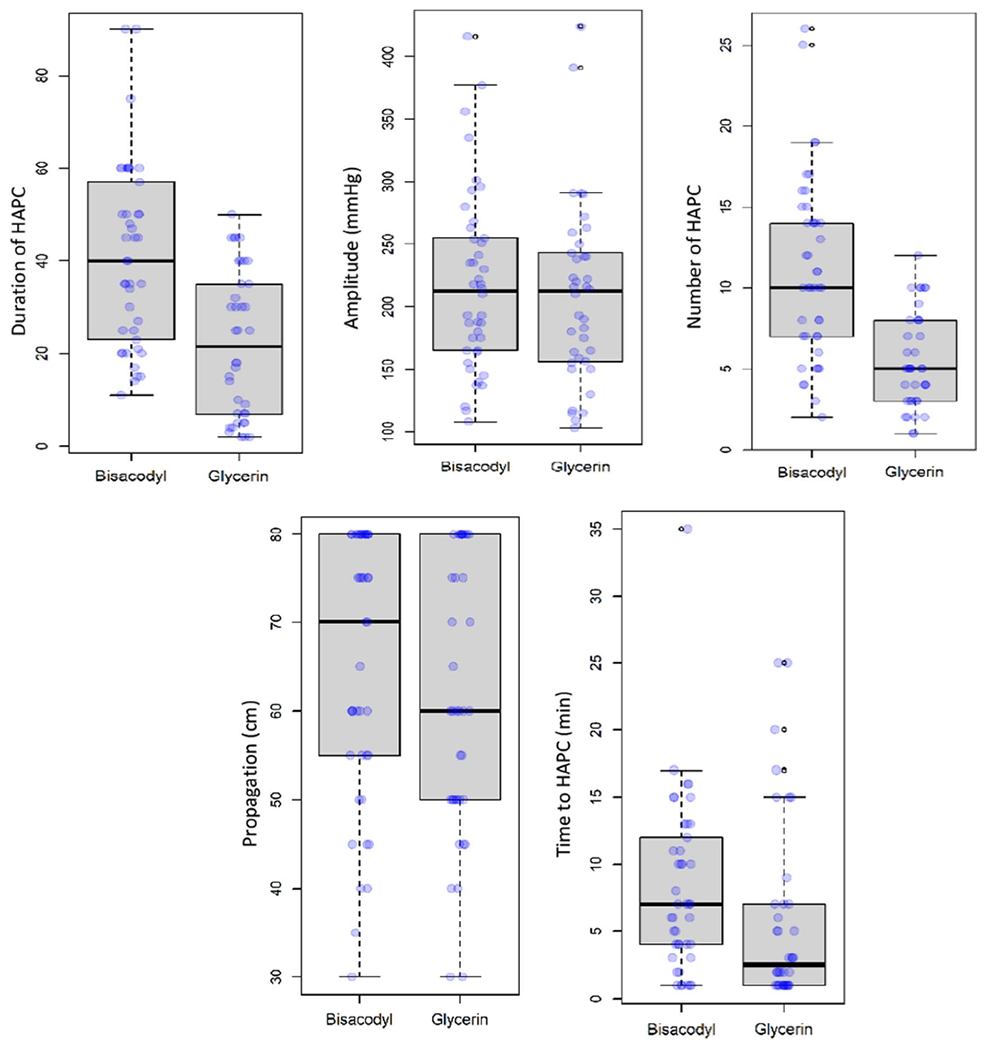
Box plots of HAPC outcome by drug (duration, amplitude, number, propagation, and time to HAPC postdrug administration). Significant differences were found in duration, number, and propagation of HAPC. HAPC, high amplitude propagated contractions.

The data were not highly skewed on density plots of HAPC characteristics so no transformation was done. After adjusting for drug sequence (i.e., study protocol variable, which represents the carry-over effect) and period effect, post-Bisacodyl HAPCs had significantly longer duration (*p* <0.0001), higher number of HAPC (*p* <0.0001), and longer propagation (*p* = 0.02) than the post-Glycerin HAPCs (see Table 2). There was inconsistent documentation of the patients’ symptoms during the manometry studies (Figure 2).

### 3.3 | Effect of baseline characteristics on HAPC findings

Age and gender did not affect any of the outcomes significantly and therefore were removed from the mixed-effect models. The indication for CM (etiology), WSCE, and anorectal manometry results did not seem to affect the characteristics of HAPC in both groups (all with insignificant interactions, data not shown). Four patients did not respond to Glycerin but responded to Bisacodyl, 3 of them had chronic idiopathic constipation and one with history of pseudo-obstruction (neuropathic pattern) with similar WSCE findings (mild colonic enlargement and redundancy).

## 4 | DISCUSSION

The pathognomonic sign of intact neuromuscular function is the presence of HAPC on CM. HAPCs can occur during the fasting state (more frequent in younger children), postprandially as a normal response to feeds (provided that there is an adequate caloric intake (at least 10 kcal/kg) or 400 kcal; >30% kcal from lipids)^20^ and poststimulation by colonic stimulants administered in the colon as an enema (antegrade or retrograde).^5,6,9^ The most commonly used medications for colonic stimulation are Bisacodyl and Glycerin, both of which induce colonic contractions and bowel movements.^11,12,22,23^ Bisacodyl is most often used in CM protocols as there have been no previous studies that compare the stimulatory effect of both medications. To our knowledge, this is the first study comparing the effect of Bisacodyl and Glycerin on HAPC parameters measured by colonic manometry.

Variability of response to stimulant laxatives has been reported in practice and may be dose dependent.^22^ In our center’s experience in patients seen in our colorectal program, it is not clear if there are patient factors, like age or etiology, that may be responsible for the variability in the response noted by our group. There is currently no data to guide clinicians as to which is more effective in our group’s experience.

Our results show that, in the same patient, Bisacodyl induced more HAPCs that last longer and propagate for a longer distance than HAPCs induced after the administration of Glycerin. This could indicate that Bisacodyl has a more potent effect on the colon in inducing bowel movements than Glycerin.^23^ Bisacodyl may be a more effective medication when used in antegrade as well as retrograde enemas in patients who do not respond well to Glycerin. In our cohort, all 4 of the patients who did not have HAPC with Glycerin had HAPCs with Bisacodyl, further highlighting the stronger effect of Bisacodyl stimulation on the colon. Three patients had similar etiology (chronic idiopathic constipation) with similar anatomical findings on WSCE and one with neuropathic dysmotility.

Our patients served as their own control regardless of the etiology of constipation; therefore, we were able to include all of our children undergoing CM making it a stronger study design which also helped power our study adequately. We also accounted for the carry-over effect of each medication so the results were not confounded. We only included patients undergoing CM without antroduodenal manometry combined studies to avoid any potential confounding effects from other medications as well as allow for adequate timing between the stimulant medications dosing during CM.

Limitation included the lack of documentation of all symptoms during the study to compare symptoms with Glycerin and Bisacodyl like abdominal pain, cramping, and emesis. None of the patients included had any severe symptoms after the administration of either drug that necessitated discontinuation of the study though. Hence, we completed our objective evaluation of HAPC poststimulation with both medications. Additionally, most of our study participants had functional (idiopathic) constipation with minimal comorbidities which did not allow us to compare HAPC in functional constipation vs other etiology though etiology and associated comorbidities on data analysis did not seem to have an effect on the results. Larger studies may be needed to run a sub-analysis on HAPC characteristics with both medications in sub-groups depending on the etiology of their symptoms. The drug doses were age dependent so we did not compare different Glycerin and Bisacodyl doses on the colon in the same patient.

## 5 | CONCLUSION

Overall, Bisacodyl seemed to be more effective than Glycerin in inducing more frequent, longer lasting HAPCs with longer propagation during CM, making Bisacodyl a potentially more effective medication for inducing bowel movements in patients with both constipation and in the treatment of encopresis with continence enemas.

## Figures and Tables

**TABLE 1:** Demographics and cohort description by protocol.

Variable	Bisacodyl 1st (*N* = 22)	Glycerin 1st (*N* = 23)	Overall (*N* = 45)	*p*-value
Age, years (Median, IQR)	9.5 (7.0, 11.0)	12.0 (9.0, 16.0)	10.0 (8.0, 13.0)	**0.02**

Gender, female (*n*, %)	10 (45.5)	9 (39.1)	19 (42.2)	0.67

BMI (Median, IQR)	15.7 (14.9, 18.0)	18.4 (15.7, 23.2)	16.5 (15.3, 19.9)	**0.02**

BMI, *Z*-score (Median, IQR)	−0.1 (−0.7, 0.9)	0.1 (−0.2, 0.7)	0 (−0.6, 0.7)	0.34

BMI, percentile (Median, IQR)	37.6 (19.4, 61.9)	52.0 (43.8, 76.2)	46.5 (27.2, 74.0)	0.11

Race (*n*, %)				
White	20 (90.9)	21 (91.3)	41 (91.1)	1
Hispanic	1 (4.5)	0	1 (2.2)	
African American	1 (4.5)	2 (8.7)	3 (6.7)	

Chronic idiopathic constipation (*n*, %)	16 (72.7)	18 (78.3)	16 (72.7)	0.67

Comorbidities (*n*, %)	8 (36.4)	12 (52.2)	20 (44.4)	0.29

ARM results (*n*, %)				
Not done	0	1 (4.3)	1 (2.2)	0.45
Normal	8 (36.4)	11 (47.8)	19 (42.2)	
Abnormal	14 (63.6)	11 (47.8)	25 (55.6)	

WSCE (*n*, %)				
Normal	7 (31.8)	6 (26.1)	13 (28.9)	0.55
Rectosigmoid dilation	7 (31.8)	5 (21.7)	12 (26.7)	
Global dilation and redundancy	8 (36.4)	12 (52.2)	20 (44.4)	

Fasting HAPC (*n*, %)	9 (40.9)	4 (17.4)	13 (28.9)	0.08

Postprandial HAPC (*n*, %)	14 (63.6)	11 (47.8)	25 (55.6)	0.29

Post-Bisacodyl HAPC (*n*, %)	22 (100)	20 (90.9)	42 (95.5)	0.49

Post-Glycerin HAPC (*n*, %)	20 (90.9)	18 (81.8)	38 (86.4)	0.66

*Note*: Bold values are the statistically significant values with *p* <0.05.

Abbreviations: ARM, anorectal manometry: HAPC, high amplitude propagated contractions; WSCE, water-soluble contrast enema.

**TABLE 2:** HAPC characteristics after the two drugs.

Variable	Stat	Post-Bisacodyl	Post-Glycerin	*p*-value*
Duration (min)	*N* ^a^	42	38	<0.0001
	Median (Q1, Q3)	40.0 (23.0, 57.0)	21.5 (7.0, 35.0)	

Highest Amplitude (mmHg)	*N*	42	38	0.14
	Median (Q1, Q3)	212.5 (165.0, 255.0)	212.0 (156.0, 243.0)	

Number of HAPC	*N*	42	38	<0.0001
	Median (Q1, Q3)	10.0 (7.0, 14.0)	5.0 (3.0, 8.0)	

Propagation (cm)	*N*	42	38	0.02
	Median (Q1, Q3)	70.0 (55.0, 80.0)	60.0 (50.0, 80.0)	

Time to HAPC (min)	*N*	42	38	0.11
	Median (Q1, Q3)	7.0 (4.0, 12.0)	2.5 (1.0, 7.0)	

a Forty two out of 45 (95.5%) patients had HAPCs after Bisacodyl; 38/45 (86.4%) had HAPCs after Glycerin.

* *p* values are from the mixed-effect model controlling for drug sequence and period.

## Data Availability

The data that support the findings of this study are openly available in Neurogastroenterology and Motility at https://onlinelibrary.wiley.com/journal.

## Practitioner Points

1. Bisacodyl is more effective than Glycerin as a colonic stimulant in inducing HAPCs.
2. Bisacodyl is potentially more effective than Glycerin in the treatment of constipation.
3. Bisacodyl may be considered as a step up therapy if there is failure/suboptimal clinical response to Glycerin.
